# Early epidural analgesia for the preoperative management of suspected spontaneous pheochromocytoma rupture: a case report

**DOI:** 10.1186/s40981-025-00813-9

**Published:** 2025-09-26

**Authors:** Yukina Oshiba, Sho C. Shibata, Taehun Lee, Junichi Inoue, Takashi Kita

**Affiliations:** 1 Department of Anesthesiology and Intensive Care Medicine, Osaka University Graduate School of Medicine, Suita, Japan; 2 Department of Anesthesiology, Osaka International Medical & Science Center, Osaka, Japan

**Keywords:** Pheochromocytoma, Epidural analgesia, Perioperative management, Abdominal pain, Preoperative optimization

## Abstract

**Background:**

Acute abdominal pain is an uncommon manifestation of pheochromocytoma. Poor pain management may delay preoperative optimization of hemodynamics and intravascular blood volume. We report a case in which early epidural analgesia facilitated preoperative preparation for a laparoscopic adrenalectomy.

**Case presentation:**

A female patient diagnosed with pheochromocytoma developed the sudden onset of severe left lower abdominal pain, hypertension, nausea, and vomiting. Pain treatment with acetaminophen and loxoprofen was insufficient, and opioids were avoided due to persistent nausea and constipation. Initiation of continuous thoracic epidural analgesia with 0.25% levobupivacaine resulted in rapid pain relief and improved gastrointestinal symptoms. Over a 9-day period, blood pressure and intravascular blood volume were optimized while epidural analgesia was continued. She underwent a successful adrenalectomy and was discharged without complications.

**Conclusions:**

This case illustrates the potential utility of early epidural analgesia in the preoperative management of pheochromocytoma, particularly in patients with refractory abdominal pain and limited opioid tolerance.

## Introduction

Pheochromocytomas are rare catecholamine-producing neuroendocrine tumors that arise from adrenal medullary chromaffin cells. The estimated global incidence rate for pheochromocytomas and paragangliomas is approximately 0.4 to 0.95 per 100,000 [1]. While clinical presentation varies, typical symptoms include episodes of headache, palpitations, and diaphoresis [2]. Gastrointestinal manifestations, such as nausea, vomiting, and constipation, are less common. A rare complication of pheochromocytoma is acute abdominal pain, which is often considered as the result of spontaneous hemorrhagic infarction within the tumor and can lead to catecholamine-induced hypertensive crisis [3, 4]. The current recommended treatment for pheochromocytoma is minimally invasive adrenalectomy, preceded by a 7-to-10-day preoperative medical treatment period. During this period, blood pressure and heart rate are normalized with alpha-adrenergic receptor blockers, such as doxazosin or phenoxybenzamine. Concurrently, catecholamine-induced blood volume contraction is corrected with a high-sodium diet and liberal fluid intake, in accordance with the established endocrine society guidelines [5]. However, episodic periods of severe abdominal pain can complicate preoperative medical treatment and pharmacological titration. Analgesic options for abdominal pain and discomfort in pheochromocytoma patients may be limited in some cases. Opioid use is complicated by pro-emetic effects, and drugs such as pethidine, tramadol, and metoclopramide may precipitate exaggerated blood pressure response [6].

In this report, we describe a case in which epidural analgesia was effective in controlling abdominal pain and facilitating preoperative hemodynamic management in a young female presenting with severe abdominal pain scheduled for laparoscopic left adrenalectomy for pheochromocytoma.

## Case presentation

A young female with a two-year history of intermittent abdominal pain was diagnosed with pheochromocytoma after undergoing evaluation. Contrast-enhanced computed tomography (CT) revealed a 40 mm diameter mass on her left adrenal gland with elevated plasma and urine metanephrines. The diagnosis was made one week prior to presentation. She had no known family history of endocrine disorders. During an outpatient consultation to discuss surgical options, she suddenly developed severe left lower quadrant abdominal pain and was transferred to the emergency department. On admission, her vital signs revealed elevated blood pressure (180/121 mmHg) and tachycardia (102 bpm). Physical examination revealed marked pain and tenderness in the left lower abdomen. Laboratory findings demonstrated an elevated inflammatory response (CRP: 9.72 mg/dL) and markedly elevated catecholamine levels: adrenaline 1.0 ng/mL, noradrenaline 4.1 ng/mL, free metanephrines 492 pg/mL, and normetanephrines 1360 pg/mL. Elevated levels of free metanephrines 2.7 mg/day and normetanephrines 3.1 mg/day in 24-h urine collection were also later noted. Further evaluation using magnetic resonance imaging (MRI) showed a T1 signal hyperintense region within the adrenal mass, suggestive of intratumoral hemorrhage and impending rupture.

The patient was admitted to the intensive care unit (ICU), restricted to bed rest, and monitored with continuous invasive arterial blood pressure and standard ICU monitoring. Due to her worsening clinical status, an adrenalectomy was expedited. After admission, her abdominal pain intensified with vomiting and nausea despite receiving oral loxoprofen (up to 180 mg/day) and acetaminophen (4000 mg/day), respectively.

She reported persistent nausea, vomiting, constipation, and inability to eat or sleep due to worsening abdominal pain. Preoperative alpha-blockade with doxazosin and phentolamine proved challenging, resulting in delayed control of hypertension and suboptimal intravascular optimization. The multidisciplinary perioperative pain management team was consulted.

Given her likely low tolerance to opioids and ongoing gastrointestinal symptoms, epidural analgesia with local anesthetics was deemed preferable. The possibility that a stress response related to epidural catheter placement could cause hypertension and increase the risk of tumor rupture was also considered. However, after confirming that the patient understood the possible risks and obtaining her consent, we proceeded with the intervention. On day 2 of ICU admission, she was transferred to the operating room for epidural catheter placement under close heart rate and blood pressure monitoring. A conscious effort was made to reduce any pain or distress to mitigate hypertension during the procedure. The epidural catheter (Perifix®, B. Braun) was inserted at the Th 7–8 level, and the epidural infusion was managed using a programmable epidural infusion pump (CADD-Solis®, Smiths Medical). The programmed regimen included a combination of intermittent bolus mode (4 mL of 0.25% levobupivacaine administered every 60 min) and patient-controlled analgesia mode (4 mL bolus with a lockout period of 60 min). After placement, a large initial bolus dose was avoided due to the risk of significant vasodilation and hypotension due to sympathetic nerve blockade.

**Fig. 1:**
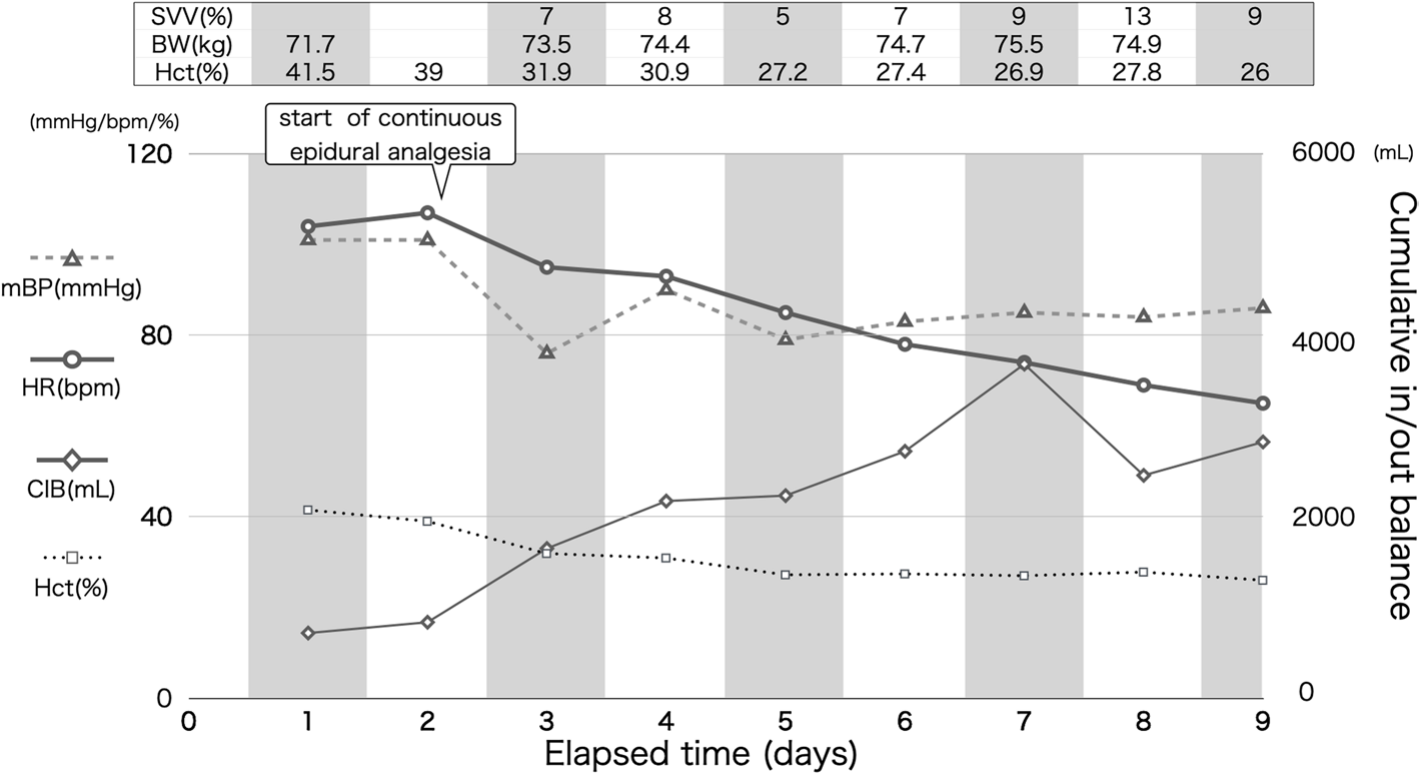
Preoperative Intensive Care Unit (ICU) Monitoring and Analgesic Effects. The patient was admitted to the ICU with continuous arterial blood pressure and standard ICU monitoring. On ICU day 2, a thoracic epidural catheter was placed, and 0.25% levobupivacaine was administered via programmed intermittent epidural bolus (4 mL every 60 min) and patient-controlled epidural analgesia (4 mL bolus, 60-min lockout). Epidural analgesia led to significant pain relief and recovery of bowel movements (BM) within 48 h. Intravascular volume management was assessed by cumulative in–out fluid balance (CIB), daily body weight, and serial stroke volume variation (SVV). Abbreviations: mBP, mean blood pressure; HR, heart rate; CIB, cumulative in–out balance; BM, bowel movement

**Fig. 2:**
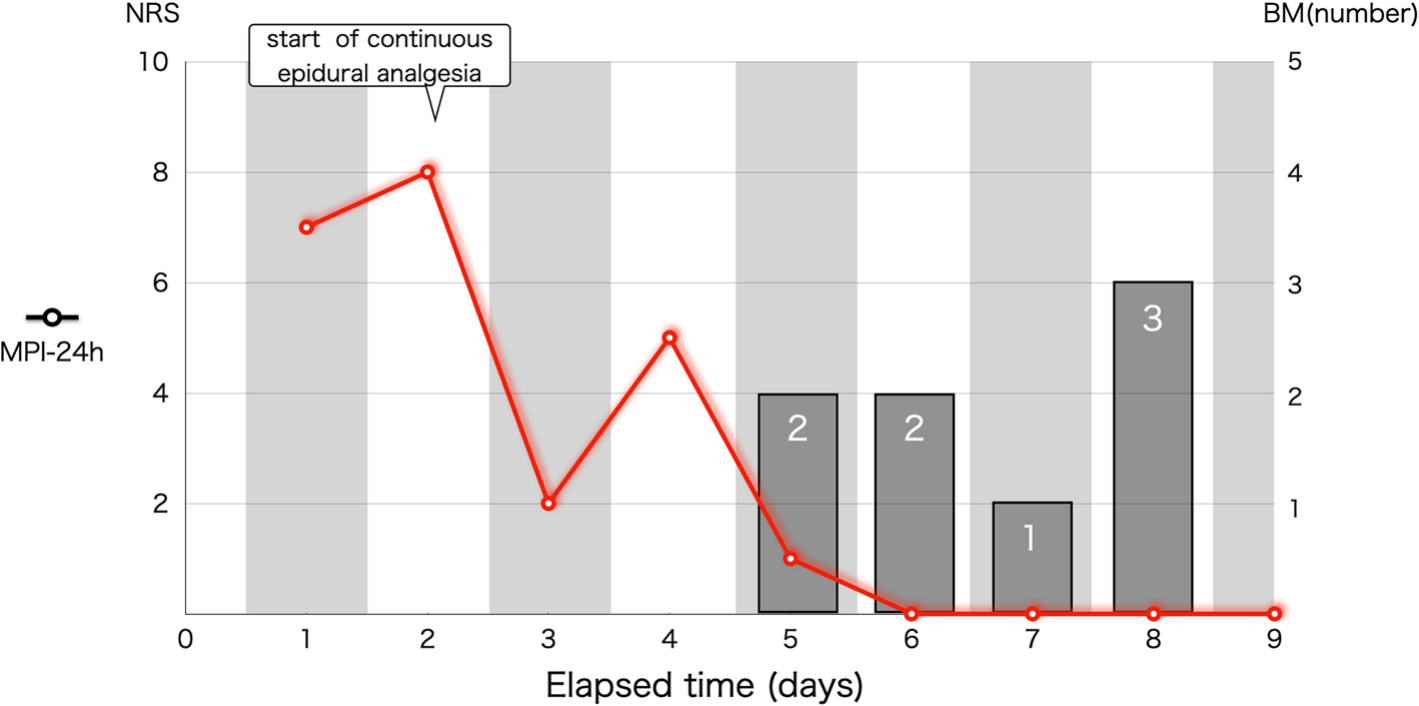
Time Course of Pain Intensity and Bowel Movements During Preoperative Period. Maximum pain intensity over 24 h (MPI-24) and frequency of bowel movements (BM, number per 24 h) are shown during the preoperative period. Initiation of thoracic epidural analgesia on day 2 resulted in a marked reduction in MPI-24 and restoration of regular BM frequency. BM frequency reflects the number of self-reported or observed events per 24 h. Abbreviations: MPI-24, maximum pain intensity over the last 24 h; BM, bowel movement

Following the initiation of thoracic epidural analgesia, a significant reduction in maximum pain intensity over 24 h was observed (Fig. 2). Symptom improvement allowed for gradual titration of oral alpha-blocker phentolamine from 2 mg/day to 8 mg/day over 9 days and in parallel with fluid replacement therapy and stabilization of hemodynamics. Trends in intravascular volume were assessed by evaluating cumulative in–out fluid balance, and daily changes in body weight. Stroke volume variation (SVV) was monitored as an additional indicator of intravascular volume status but was interpreted with caution, as its reliability is limited in spontaneously breathing patients. SVV trends were considered alongside cumulative in–out fluid balance, daily body weight changes, and overall clinical assessment, rather than being used as a sole determinant for fluid management (Fig. 1). Recovery of bowel movements was observed after 48 h, with multiple bowel movements (BM) recorded by ICU staff and expressed as the number of BMs per 24 h (Fig. 2). The frequency of BM was determined primarily by the patient’s self-reporting, as the patient remained fully conscious and alert throughout the preoperative period.

After nine days of optimization in the ICU, she underwent laparoscopic left adrenalectomy under combined epidural and general anesthesia. Anesthesia was maintained with sevoflurane, remifentanil, and a bolus infusion of 0.25% levobupivacaine from the epidural catheter. The surgery proceeded uneventfully, and blood pressure remained stable throughout adrenal vein clipping and tumor resection. Dobutamine at 3 μg/kg/min was started after tumor removal but was discontinued postoperatively. She was extubated shortly after the end of surgery and transferred back to the ICU. Postoperative analgesia was maintained with scheduled acetaminophen and continuous epidural infusion of 0.25% levobupivacaine (4 mL/h), together with dexmedetomidine (25 μg/h) for sedation. Hemodynamic stability and satisfactory pain control were maintained, and the epidural catheter was removed on postoperative day 2. The patient was discharged on postoperative day 10 without complications.

## Discussion

Pheochromocytomas are rare catecholamine-producing neuroendocrine tumors originating from the adrenal medulla, with an estimated incidence of approximately 0.6 per 100,000 individuals annually [1]. While the classic symptoms include episodic headaches, palpitations, hypertension, and diaphoresis [2], patients may also present with nonspecific symptoms such as anxiety, tremors, chest or abdominal pain, nausea, vomiting, diarrhea, and constipation. If left untreated, pheochromocytomas can progress to serious complications including cardiomyopathy, cerebral hemorrhage, and pulmonary edema [4, 7]

Severe, acute abdominal pain, although uncommon (reported in 14% of cases), can complicate the diagnostic and preoperative management process [8]. This pain is most often attributed to tumor mass effect, catecholamine-induced gastrointestinal dysmotility, or, more rarely, spontaneous tumor rupture and hemorrhage, which may precipitate a hypertensive crisis and systemic instability [3, 9, 10].

In the current case, early cross-sectional imaging and biochemical evaluation enabled prompt diagnosis and supported a controlled, expedited preoperative strategy. MRI revealed limited intratumoral hemorrhage in the absence of hemodynamic collapse, which prompted stabilization and timely surgical intervention.

Analgesic management posed a particular challenge: standard non-opioid agents, including acetaminophen and loxoprofen, proved inadequate. Opioid analgesics, including intravenous fentanyl, were avoided owing to concerns that they would worsen the patient’s nausea, vomiting, and constipation and further delay gastrointestinal recovery. While fentanyl is recognized for perioperative analgesia [11], the risk of opioid-induced emesis leading to sympathetic surges and potential tumor rupture outweighed its benefits. Dopamine antagonist antiemetics, such as metoclopramide, were used sparingly due to their potential to provoke catecholamine release [6]. Ondansetron was not available through our health system. These factors supported the early use of epidural analgesia and the decision to avoid opioids in this case.

Although epidural anesthesia is well established for intraoperative and postoperative pain control in pheochromocytoma resection, its role in the preoperative period has been rarely reported. In this case, early initiation of thoracic epidural analgesia during preoperative optimization provided rapid and sustained pain relief, improved gastrointestinal symptoms, and facilitated titration of alpha-blockers and fluid management. These effects contributed to hemodynamic stability and allowed adequate preparation for adrenalectomy.

Placement of an epidural catheter in a patient with active catecholamine excess carries potential risks, including the possibility of transient hypertension during insertion and pronounced hypotension after sympathetic blockage-particularly in states of intravascular dehydration. To minimize such fluctuations, a large initial bolus was deliberately avoided, and dosing was carefully titrated. This cautious approach led to effective analgesia without significant hemodynamic compromise.

The use of SVV for fluid optimization in this case merits further comment. Although dynamic indices, such as SVV, can inform intravascular volume assessment, their accuracy decreases when patients are not mechanically ventilated due to irregular intrathoracic pressure changes [12]. For this reason, we used SVV as a supplemental tool, placing primary reliance on cumulative fluid balance, daily weight monitoring, hematocrit changes, and the patient’s hemodynamic and clinical response for volume management decisions.

Early use of continuous epidural analgesia may provide not only symptomatic relief but also physiological benefits, such as reduced systemic vascular resistance and increased venous capacitance. These effects can facilitate intravascular volume expansion and may prevent severe hypotension following tumor removal. Previous reports of epidural analgesia in similar contexts are limited [13, 14]. For instance, Okumura et al. describe epidural analgesia use for postoperative intestinal pseudo-obstruction in pheochromocytoma but did not observe improved bowel recovery when initiated intraoperatively [15]. In contrast, the current case demonstrates that continuous epidural analgesia initiated in the preoperative period, at higher local anesthetic concentrations, was associated with recovery of BMs within 48 h. The difference between reports may relate to timing, severity of gastrointestinal symptoms, or procedural details. Further studies are needed to clarify the precise role and indications for early epidural analgesia as a strategy to improve gastrointestinal function or facilitate hemodynamic optimization in pheochromocytoma. Based on the available evidence, the decision to pursue preoperative epidural analgesia should be individualized, considering patient symptoms, volume status, and risk for adverse events.

Compared to the report by Kurdi et al., where epidural infusion was initiated only 48 h preoperatively primarily for blood pressure control, our prolonged, preoperative use of thoracic epidural analgesia over 9 days was aimed chiefly at analgesia and symptom relief [16]. This case supports the potential benefit of extended epidural analgesia in improving preoperative patient comfort and aiding in hemodynamic stabilization when conventional analgesics are poorly tolerated.

In summary, this report describes successful preoperative management of pheochromocytoma-associated refractory abdominal pain using early and continuous epidural analgesia, which contributed to pain relief, restoration of bowel function, and stable perioperative hemodynamics. While further evidence is needed, early preoperative epidural analgesia may serve as a valuable adjunct in the multidisciplinary management of pheochromocytoma in select, symptomatic patients.

## Data Availability

Data sharing is not applicable to this article as no datasets were generated or analyzed during the current study.

## Abbreviations

BM: Bowel movement
mBP: Mean blood pressure
HR: Heart rate
SVV: Stroke volume variation
ICU: Intensive care unit
